# Supplementary material for: Reducing the risks of nuclear war: the role of health professionals
Source: Eur J Public Health. 2023 Aug 25;33(5):755–6. doi: 10.1093/eurpub/ckad150 (PMC10567236; doi:10.1093/eurpub/ckad150)
Supplement: ckad150_Supplementary_Data [file ckad150_supplementary_data.docx]

Supplementary Data

1. Lewis P, Williams H, Pelopidas, Aghlani S. Too close for comfort, cases of near nuclear use and options for policy. Chatham House Report. April, 2014. <https://www.chathamhouse.org/2014/04/too-close-comfort-cases-near-nuclear-use-and-options-policy> (accessed June 1, 2023).
2. Bivens M. Nuclear famine. IPPNW. August, 2022. <https://www.ippnw.org/wp-content/uploads/2022/09/ENGLISH-Nuclear-Famine-Report-Final-bleed-marks.pdf> (accessed June 1, 2023).
3. Xia L, Robock A, Scherrer K, et al. Global food insecurity and famine from reduced crop, marine fishery and livestock production due to climate disruption from nuclear war soot injection. *Nat Food* 2022; **3:** 586–96.
4. Helfand I, Lewis P, Haines A. Reducing the risks of nuclear war to humanity. *Lancet* 2022; **399:** 1097–98.
5. Nobel Prize Outreach AB. International Physicians for the Prevention of Nuclear War—facts. 1985. <https://www.nobelprize.org/prizes/peace/1985/physicians/facts/> (accessed June 1, 2023).
6. UN Office for Disarmament Affairs. Treaties Database. Treaty on the Prohibition of Nuclear Weapons, status of the Treaty. 2023. <https://treaties.unoda.org/t/tpnw> (accessed June 1, 2023).
7. Center for Arms Control and Non-Proliferation. No first use: frequently asked questions. 2023. <https://armscontrolcenter.org/issues/no-first-use/no-first-use-frequently-asked-questions/>  (accessed  June 2, 2023).
